# Supplementary material for: Healthcare utilization in children across the care continuum during the COVID-19 pandemic
Source: PLoS One. 2022 Oct 27;17(10):e0276461. doi: 10.1371/journal.pone.0276461 (PMC9612476; doi:10.1371/journal.pone.0276461)
Supplement: S1 File — (PDF) [file pone.0276461.s001.pdf]

Supplementary Table 1: Demographic trends comparing early and mid-pandemic to pre-pandemic periods

	Early Pandemic*		Mid-Pandemic*	
	Change in % of pediatric Optum pop.	P-value	Change in % of pediatric Optum pop.	P-value
**Female sex**	0.0% [-0.0%, 0.0%]	0.1	0% [-0.0%, 0.0%]	0.32
**Age (years)**				
< 1	0.0% [-0.1%, 0.0%]	0.4	-0.1% [-0.1%, -0.0%]	0.02
1 – 5	-0.3% [-0.4%, -0.1%]	0.0001	-0.3% [-0.5%, -0.1%]	0.003
6 – 12	0.1% [-0.0%, 0.1%]	0.05	0.1% [0.0%, 0.2%]	0.01
13 – 17	0.3% [0.1%, 0.4%]	0.005	0.3% [0.0%, 0.5%]	0.03
**Race/Ethnicity** (removing unknown)				
non-Hispanic White	0.1% [-0.1%, 0.4%]	0.3	0.2% [-0.2%, 0.5%]	0.32
non-Hispanic Black	0.0% [-0.2%, 0.1%]	0.83	0.0% [-0.2%, 0.2%]	0.95
Hispanic	0.1% [-0.0%, 0.2%]	0.05	0.0% [-0.2%, 0.1%]	0.73
Asian	-0.2% [-0.4%, -0.1%]	0.0002	-0.1% [-0.3%, 0.0%]	0.11
**Census Region**				
New England	-0.1% [-0.2%, -0.0%]	0.01	-0.1% [-0.2%, 0.0%]	0.19
Middle Atlantic	-0.1% [-0.2%, -0.0%]	0.01	-0.2% [-0.4%, -0.1%]	0.004
East North Central	1.1% [0.6%, 1.6%]	0.005	1.1% [0.5%, 1.7%]	0.0001
West North Central	-0.2% [-0.4%, 0.0%]	0.11	-0.6% [-1.0%, -0.2%]	0.004
South Atlantic	-1.0% [-1.5%, -0.5%]	0.0001	-0.7% [-1.4%, -0.1%]	0.03
East South Central	-0.1% [-0.1%, -0.0%]	0.002	0.1% [0.0%, 0.2%]	0.01
West South Central	0.6% [-0.9%, 2.0%]	0.44	0.6% [-1.2%, 2.3%]	0.53
Mountain	-0.1% [-0.5%, 0.2%]	0.39	-0.3% [-0.7%, 0.1%]	0.1
Pacific	0.7% [0. 3%, 1.1%]	0.001	1.2% [0.6%, 1.8%]	< 0.0001
**Income quartile** (of patient zip code)				
Lowest (< $55,019)	-0.2% [-0.5%, 0.1%]	0.24	-0.5% [-0.9%, -0.1%]	0.03
Second ($55,020 - $72,759)	0.1% [0.0%, 0.2%]	0.02	0.0% [-0.2%, 0.1%]	0.61
Third ($72,759 - $96,969)	0.1% [0.0%, 0.3%]	0.01	0.3% [0.2%, 0.4%]	< 0.0001
Highest (> $96,969)	0.0% [-0.3%, 0.2%]	0.73	0.2% [-0.2%, 0.6%]	0.26

*Changes calculated using an interrupted time series approach; Early pandemic = mid-March 2020 – mid-June 2020 and Mid-pandemic = mid-June 2020 through March 2021

Supplementary Figure 1: Trends in race/ethnicity over time, with horizontal dotted lines representing the start of the early and mid-pandemic periods

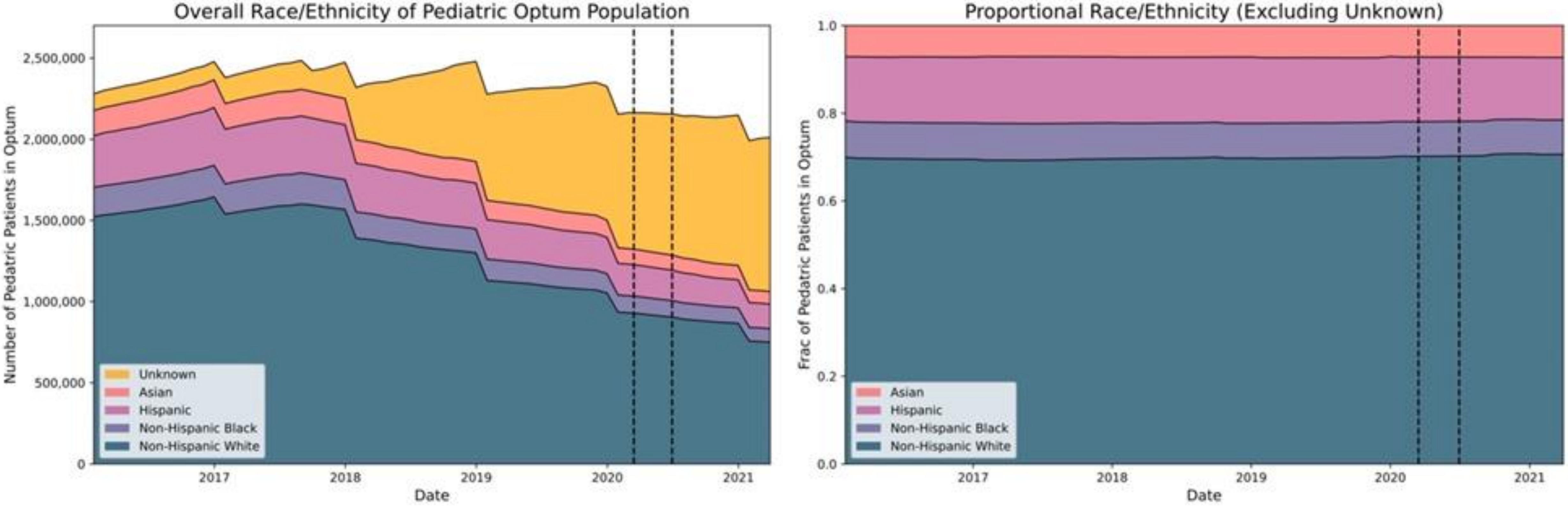

Supplementary Figure 2: Trends in pre-pandemic top 7 causes of hospitalization in non-psychiatric facilities, with dotted lines representing starts of early and mid-pandemic periods

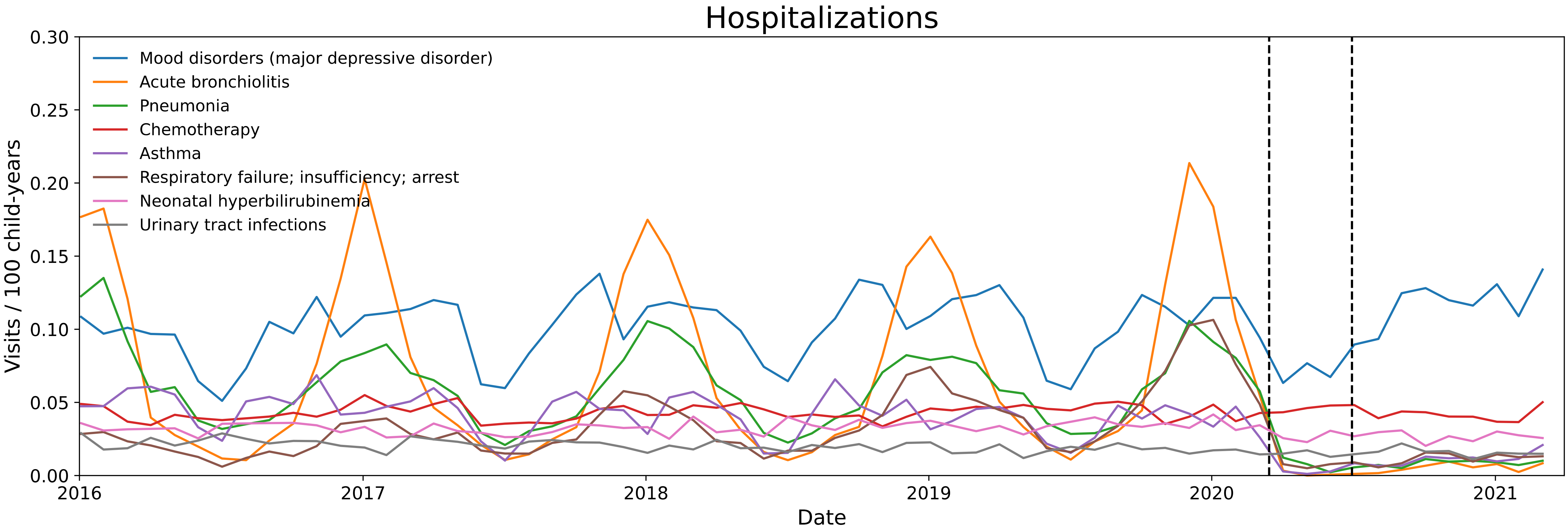

Supplementary Figure 3: Pandemic changes in leading causes of PCP sick visits with heat map

Diagnosis	Rank Pre-Pandemic	Rank Post-Pandemic	% Change during early pandemic*	% Change during mid-pandemic*
Streptococcal sore throat	1	3	-84.8% [-87.8% , -81.1%]	-66.1% [-72.0% , -58.9%]
Otitis media	2	6	-85.2% [-88.1% , -81.6%]	-76.9% [-82.5% , -69.3%]
Acute upper respiratory infection	3	5	-91.1% [-94.2% , -86.6%]	-71.3% [-77.6% , -63.4%]
Viral infection	4	9	-68.8% [-73.7% , -63.1%]	-58.7% [-66.5% , -49.2%]
Fever of unknown origin	5	8	-63.0% [-69.4% , -55.3%]	-57.0% [-72.1% , -33.7%]
Attention-deficit hyperactivity disorder	6	2	-26.7% [-28.4% , -24.9%]	-16.3% [-20.6% , -11.8%]
Cough	7	12	-75.8% [-81.3% , -68.8%]	-65.7% [-72.1% , -57.8%]
Allergic reactions	8	7	-25.7% [-30.7% , -20.4%]	-12.8% [-18.2% , -6.9%]
Acute sinusitis	9	30	-88.4% [-92.2% , -82.9%]	-78.5% [-82.3% , -73.9%]
Medical examination/evaluation	10	4	-50.7% [-57.1% , -43.4%]	-12.9% [-19.2% , -6.2%]
Inflammation; infection of eye (except caused by TB or STD)	11	24	-74.2% [-76.2% , -72.0%]	-68.4% [-73.4% , -62.6%]
Influenza	12	113	-98.3% [-99.1% , -96.9%]	-97.6% [-99.4% , -90.7%]
Asthma	13	21	-61.0% [-66.6% , -54.4%]	-59.0% [-65.0% , -52.0%]
Other ear and sense organ disorders	14	10	-33.9% [-39.0% , -28.3%]	-18.3% [-35.4% , 3.3%]
Allergic rhinitis	15	17	-66.4% [-70.5% , -61.7%]	-30.3% [-38.7% , -20.6%]
Cellulitis	16	14	-26.1% [-29.0% , -23.0%]	-26.1% [-30.4% , -21.6%]
Abdominal pain	17	16	-58.2% [-63.7% , -51.9%]	-18.9% [-26.1% , -10.9%]
Common cold	18	45	-91.5% [-94.4% , -86.9%]	-69.9% [-76.8% , -61.0%]
Croup	19	53	-94.5% [-96.6% , -91.1%]	-81.8% [-87.4% , -73.8%]
Feeding difficulties and mismanagement	20	13	-16.5% [-19.2% , -13.7%]	-10.9% [-16.0% , -5.5%]
Other upper respiratory disease	21	22	-79.2% [-83.0% , -74.6%]	-33.8% [-47.4% , -16.5%]
Genitourinary symptoms and ill-defined conditions	22	18	-28.8% [-32.8% , -24.6%]	-4.5% [-12.0% , 3.7%]
Other injuries and conditions due to external causes	23	25	-58.4% [-62.3% , -54.0%]	-26.5% [-36.1% , -15.5%]
Other aftercare	24	23	-51.5% [-53.7% , -49.2%]	-42.3% [-46.7% , -37.6%]
Other skin disorders	25	19	-28.4% [-35.1% , -21.2%]	-5.2% [-16.5% , 7.5%]
Rashes	26	20	-28.5% [-33.5% , -23.2%]	-20.8% [-25.3% , -16.1%]
Other non-traumatic joint disorders	27	27	-71.8% [-75.9% , -67.1%]	-27.2% [-36.4% , -16.7%]
Pneumonia	28	99	-92.4% [-95.3% , -87.8%]	-96.4% [-97.1% , -95.5%]
Acute bronchiolitis	29	97	-94.0% [-96.3% , -90.3%]	-92.9% [-94.9% , -90.2%]
Superficial injury; contusion	30	29	-40.1% [-44.9% , -34.9%]	-20.4% [-28.8% , -11.0%]
Headache; including migraine	32	28	-62.1% [-66.3% , -57.4%]	-15.6% [-22.8% , -7.6%]
Immunizations and screening for infectious disease	34	1	36.9% [1.1% , 85.4%]	501.3% [397.0% , 627.5%]
Anxiety disorders	37	15	-22.1% [-25.7% , -18.3%]	1.8% [-5.7% , 9.8%]
Constipation	39	26	-34.7% [-41.5% , -27.2%]	-2.4% [-12.3% , 8.7%]
Viral Infection (COVID-19)	-	11	N/A	

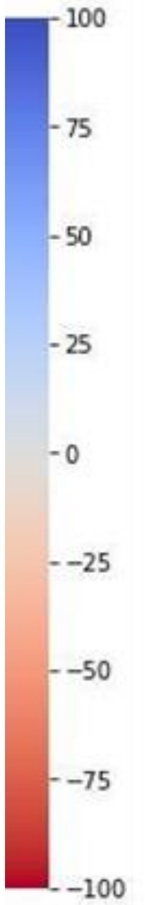

*Changes calculated using an interrupted time series approach; Early pandemic = mid-March 2020 – mid-June 2020 and Mid-pandemic = mid-June 2020 through March 2021

Supplementary Figure 4: Pandemic changes in leading causes of ED visits with heat map

Diagnosis	Rank Pre-Pandemic	Rank Post-Pandemic	% Change during early pandemic*	% Change during mid-pandemic*
Open wounds of head; neck; and trunk	1	1	-26.1% [-33.8% , -17.5%]	-15.8% [-20.8% , -10.6%]
Superficial injury; contusion	2	3	-58.3% [-64.2% , -51.5%]	-32.6% [-40.7% , -23.5%]
Abdominal pain	3	5	-65.3% [-69.8% , -60.1%]	-39.5% [-45.0% , -33.4%]
Sprains and strains	4	8	-74.5% [-78.9% , -69.2%]	-37.6% [-44.4% , -30.0%]
Fracture of upper limb	5	2	-45.6% [-51.8% , -38.6%]	-21.8% [-29.5% , -13.3%]
Fever of unknown origin	6	9	-70.2% [-73.8% , -66.2%]	-66.8% [-75.8% , -54.4%]
Acute upper respiratory infection	7	19	-88.0% [-91.8% , -82.2%]	-75.3% [-81.2% , -67.6%]
Otitis media	8	29	-84.4% [-86.5% , -82.1%]	-75.4% [-82.7% , -65.2%]
Streptococcal sore throat	9	22	-75.7% [-79.7% , -70.9%]	-62.0% [-71.2% , -49.9%]
Other bone disease and musculoskeletal deformities	10	7	-54.5% [-60.1% , -48.2%]	-30.4% [-39.4% , -20.0%]
Nausea and vomiting	11	20	-82.9% [-85.6% , -79.7%]	-61.5% [-68.6% , -52.8%]
Open wounds of extremities	12	6	-7.9% [-18.2% , 3.6%]	-2.7% [-11.4% , 6.7%]
Other injuries and conditions due to external causes	13	10	-64.7% [-69.2% , -59.5%]	-41.4% [-47.2% , -35.0%]
Allergic reactions	14	13	-66.2% [-71.6% , -59.7%]	-37.4% [-43.2% , -31.1%]
Spondylosis; intervertebral disc disorders; other back problems	15	11	-59.0% [-62.6% , -55.0%]	-25.3% [-30.6% , -19.6%]
Viral infection	16	21	-76.9% [-80.4% , -72.6%]	-66.0% [-72.6% , -57.9%]
Head injury, unspecified	17	14	-61.2% [-65.5% , -56.4%]	-35.4% [-42.6% , -27.3%]
Croup	18	38	-93.9% [-95.8% , -91.2%]	-77.1% [-83.9% , -67.4%]
Asthma	19	34	-85.6% [-88.3% , -82.3%]	-68.8% [-74.0% , -62.6%]
Influenza	20	121	-98.0% [-99.1% , -95.6%]	-98.1% [-99.7% , -88.1%]
Headache; including migraine	21	23	-70.8% [-74.5% , -66.6%]	-40.8% [-47.9% , -32.7%]
Acute bronchiolitis	22	68	-93.6% [-95.4% , -91.1%]	-92.1% [-94.9% , -87.8%]
Other non-traumatic joint disorders	23	17	-73.7% [-78.3% , -68.1%]	-45.4% [-51.3% , -38.7%]
Pneumonia	24	56	-86.1% [-90.8% , -79.2%]	-90.1% [-92.9% , -86.1%]
Cellulitis	25	30	-48.4% [-53.8% , -42.3%]	-39.8% [-45.6% , -33.4%]
Constipation	26	24	-55.2% [-65.9% , -41.2%]	-30.2% [-39.2% , -20.0%]
Concussion w or w/o loss of consciousness	27	32	-67.8% [-71.9% , -63.1%]	-37.2% [-48.6% , -23.3%]
Urinary tract infections	28	25	-42.9% [-48.4% , -36.8%]	-21.6% [-28.1% , -14.6%]
Other connective tissue disease	29	26	-63.8% [-70.6% , -55.4%]	-43.3% [-50.5% , -35.1%]
Fracture of lower limb	30	18	-35.9% [-42.1% , -29.0%]	-13.0% [-21.9% , -3.0%]
Medical examination/evaluation	31	15	-57.1% [-70.6% , -37.5%]	1.0% [-32.8% , 52.0%]
Joint disorders and dislocations; trauma-related	32	27	-42.4% [-47.1% , -37.1%]	-16.8% [-24.0% , -9.0%]
Suicide and intentional self-inflicted injury	33	16	-45.4% [-50.7% , -39.4%]	5.6% [-8.7% , 22.1%]
Mood disorders (major depressive disorder)	35	28	-50.2% [-58.9% , -39.7%]	-8.7% [-22.6% , 7.7%]
Immunizations and screening for infectious diseases	43	4	112.6% [16.4% , 288.4%]	372.9% [113.3% , 948.2%]
Viral Infection (COVID-19)	-	12	N/A	

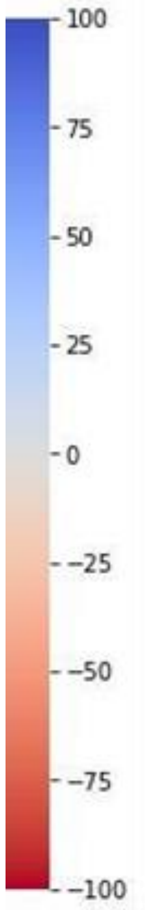

*Changes calculated using an interrupted time series approach; Early pandemic = mid-March 2020 – mid-June 2020 and Mid-pandemic = mid-June 2020 through March 2021

Supplementary Figure 5: Trends in telehealth visits over time, with dotted lines representing starts of early and mid-pandemic periods

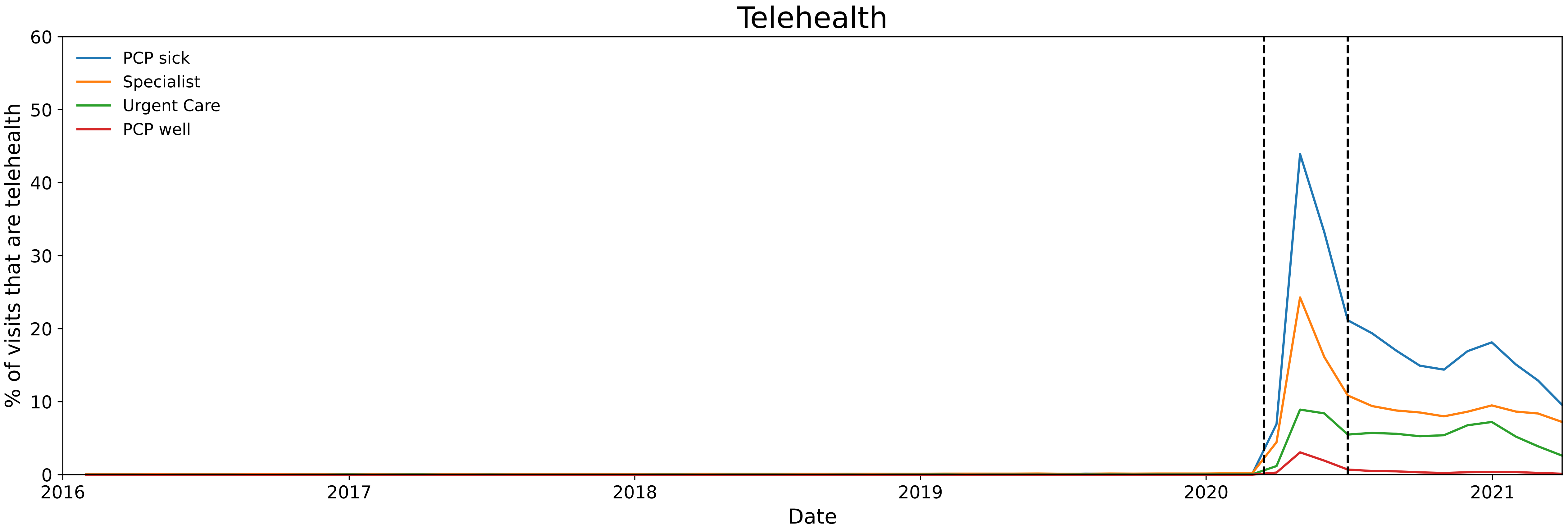

Supplementary Figure 6: Sensitivity analysis portraying pandemic changes in healthcare utilization with adjustment for age, sex, race, census region, and income quartile

Feature	Pre-pandemic count / 100 child-years	% change during early pandemic 3/15/20 – 6/15/20	% change during mid- pandemic 6/15/20 – 4/1/21
PCP well visit	95.6	-41.5% [-49.3% , -32.4%]	-7.5% [-15.5% , 1.3%]
PCP sick visit	165.0	-63.1% [-66.1% , -59.8%]	-39.1% [-46.6% , -30.5%]
ED	39.4	-61.4% [-66.3% , -55.9%]	-37.9% [-46.3% , -28.3%]
Urgent Care	20.1	-78.7% [-82.3% , -74.4%]	-37.0% [-47.5% , -24.4%]
Specialist	156.1	-46.5% [-51.1% , -41.4%]	-20.1% [-24.8% , -15.1%]
Hospitalizations	1.3	-45.2% [-51.1% , -38.5%]	-26.3% [-35.6% , -15.7%]
with ICU	0.3	-45.4% [-51.2% , -38.8%]	-29.1% [-39.4% , -17.1%]
without ICU	1.1	-45.1% [-51.4% , -38.1%]	-25.7% [-35.1% , -15.1%]
Admission to Psychiatric facilities	0.4	-42.4% [-47.1% , -37.3%]	-16.1% [-22.5% , -9.1%]
Prescriptions	288.9	-37.8% [-42.0% , -33.3%]	-25.9% [-33.6% , -17.3%]
first time	226.2	-47.7% [-51.8% , -43.3%]	-30.5% [-39.2% , -20.5%]
refills	62.7	-3.2% [-11.8% , 6.4%]	-12.7% [-18.2% , -6.8%]
