# Supplementary material for: Healthcare utilization in children across the care continuum during the COVID-19 pandemic
Source: PLoS One. 2022 Oct 27;17(10):e0276461. doi: 10.1371/journal.pone.0276461 (PMC9612476; doi:10.1371/journal.pone.0276461)
Supplement: S2 File — (DOCX) [file pone.0276461.s002.docx]

**Supplementary Material (Methods)**

This supplementary note provides additional methodology for categorization of utilization types within the Optum dataset and additional details about the interrupted time series model.

*Utilization Types*

Our classification of utilization types within the Optum dataset closely followed previous work.^1-3^

First, we identified ED and urgent care visits: ED visits, by the Place of Service (POS) code of 23 or the CPT code corresponding to the ED (99281-99288); urgent care visits, by the POS code of 20 or the provider category of “urgent care”. We also excluded CPT codes corresponding to chiropractor’s offices (98940-98942), which are occasionally misclassified in Optum with POS code 23. Of the remaining claims, office visits were identified by the CPT code (99381-99387; 99391-99397; 99210-99215); the office visits that correspond to either primary care physicians or specialists are identified by provider category. Primary care provider office visits were sorted into well visits and sick visits by CPT code (99210-99215 are for sick visits, the rest are for well visits). We identified hospitalizations by length of stay of 1+ day and excluded rehab/skilled nursing facilities and birth hospitalizations (CPT code for birth of Z381-Z388 and a patient age < 1 year). We categorized hospitalizations as psychiatric or non-psychiatric based on the hospital designation, and we categorized ICU visits using revenue codes (0200-0209). Lastly, we identified telehealth visits using either POS code of 2, or by CPT code (99441-99449; or modifier GT, GQ, 95).

*Interrupted Time Series Model*

The Interrupted Time Series (ITS) model we used was defined in two steps. First, we transformed each time series to make it roughly stationary during the pre-pandemic period. We did this by taking a logarithm and by applying yearly differencing, where you subtract off the value of the time series shifted by one year, which removes much of the seasonal effects. Second, we an ordinary least squares regression model with 4 parameters: an intercept and slope (to model the pre-pandemic trend), and two step changes for mid-March and mid-June, 2020 (to model the changes during the pandemic). The final regression model structure was:

$\ln y\left( t \right)-\ln y(t-1 \mathrm{year})\sim\alpha+\beta_{1}t+\beta_{2}I_{early pand.}+ \beta_{3}I_{late pand.}$,

where $I_{early pand.}$ is an indicator variable for months in the early pandemic period, and $I_{late pand.}$ is the same for the late pandemic. Because March 2020 was coded as being 50% pre-pandemic and 50% during the pandemic,^4^ it was coded with $I_{early pand.}=1/2.$

The output of the model is the percent change in the rate of visits during the early pandemic and middle pandemic. We used autocorrelation-robust sandwich estimators for evaluate the 95% confidence intervals.^5^

Lastly, as a sensitivity analysis, we included demographic covariates (age, sex, and race, census region, and income quartile) in the ITS model to examine the extent to which adjustment for these variables led affected the unadjusted rates.

**Supplemental References**

1. Poon SJ, Schuur JD, Mehrotra A. Trends in Visits to Acute Care Venues for Treatment of Low-Acuity Conditions in the United States From 2008 to 2015. *JAMA Intern Med*. Oct 1 2018;178(10):1342-1349. doi:10.1001/jamainternmed.2018.3205

2. Ray KN, Shi Z, Ganguli I, Rao A, Orav EJ, Mehrotra A. Trends in Pediatric Primary Care Visits Among Commercially Insured US Children, 2008-2016. *JAMA Pediatr*. Apr 1 2020;174(4):350-357. doi:10.1001/jamapediatrics.2019.5509

3. Schweiberger K, Patel SY, Mehrotra A, Ray KN. Trends in Pediatric Primary Care Visits During the Coronavirus Disease of 2019 Pandemic. *Acad Pediatr*. Nov-Dec 2021;21(8):1426-1433. doi:10.1016/j.acap.2021.04.031

4. Bernal JL, Cummins S, Gasparrini A. Interrupted time series regression for the evaluation of public health interventions: a tutorial. *Int J Epidemiol*. Feb 1 2017;46(1):348-355. doi:10.1093/ije/dyw098

5. Newey WK, West KD. A simple, positive semi-definite, heteroskedasticity and autocorrelation consistent covariance matrix. *Econometrica*. 1986;
