# Supplementary material for: Healthcare utilization in children across the care continuum during the COVID-19 pandemic
Source: PLoS One. 2022 Oct 27;17(10):e0276461. doi: 10.1371/journal.pone.0276461 (PMC9612476; doi:10.1371/journal.pone.0276461)
Supplement: Attachment — Submitted filename: Comments PON_ June 2022.docx [file pone.0276461.s003.docx]

**Healthcare Utilization in Children Across the Care Continuum During the COVID-19 Pandemic**

Overall:

The paper focused on the essential topic of “ utilization of pediatric services during Covid”. It is week written.

**Introduction:**

The information included in the second paragraph does not have any reference. In the second paragraph, “Major shifts in health care use and diagnosis patterns are important to describe as we monitor child health following the pandemic” needs to be rephrased. What shift the authors are referring to is unclear. How the training can help the shift has also not been clarified. The statement “how viral transmission and healthcare utilization impact the incidence and outcomes of specific diseases” is beyond the scope of this study as the source of data is insurance claim administrative data.

**Method**

The major concern is why include four years of data for comparison rather than taking previous year's data (March 2019-Feb 2020) for analysis? There was no justification given. The authors focused on primary care, urgent care, and ED visits, hospitalizations, and prescription medication claim but did not provide the operational definition of those terms.

**Results**

It would have been useful for the policymakers to show the utilization rate change according to the income quintile, race, and gender.

**Discussion**

The utilization rate over four years period might create a bias toward utilization rate. The conclusion about future policy or strategy the differentiation if any, across gender, race, and income quintile would have been helpful.
